# Bronchial wall T2w MRI signal as a new imaging biomarker of severe asthma

**DOI:** 10.1186/s13244-025-01939-1

**Published:** 2025-03-25

**Authors:** Ilyes Benlala, Gaël Dournes, Pierre-Olivier Girodet, François Laurent, Wadie Ben Hassen, Fabien Baldacci, Baudouin Denis De Senneville, Patrick Berger

**Affiliations:** 1 University Bordeaux, INSERM, CRCTB, U 1045, Bordeaux, France; 2 CHU de Bordeaux, Service d’imagerie Cardiaque et Thoracique, CIC-P 1401, Service d’Explorations Fonctionnelles Respiratoires, Bordeaux, France; 3 Siemens HealthCare, Erlangen, Germany; 4 LaBRI, CNRS, Bordeaux INP, UMR 5800, Bordeaux INP, UMR 5251, Talence, France; 5 Mathematical Institute of Bordeaux (IMB), University Bordeaux, CNRS, INRIA, Bordeaux INP, UMR 5251, Talence, France

**Keywords:** Asthma, MRI, Inflammation

## Abstract

**Objectives:**

Severe asthma patients are prone to severe exacerbations with a need of hospital admission increasing the economic burden on healthcare systems. T2w lung MRI was found to be useful in the assessment of bronchial inflammation. The main goal of this study is to compare quantitative MRI T2 signal bronchial intensity between patients with severe and non-severe asthma.

**Methods:**

This is an ancillary study of a prospective single-center study (NCT03089346). We assessed the mean T2 intensity MRI signal of the bronchial wall area (BrWall_T2-MIS) in 15 severe and 15 age and sex-matched non-severe asthmatic patients. They also have had pulmonary function tests (PFTs), fractional exhaled nitric oxide (FeNO) and blood eosinophils count (Eos). Comparisons between the two groups were performed using Student’s *t*-test. Correlations were assessed using Pearson coefficients. Reproducibility was assessed using intraclass correlation coefficient and Bland-Altman analysis.

**Results:**

BrWall_T2-MIS was higher in severe than in non-severe asthma patients (74 ± 12 vs 49 ± 14; respectively *p* < 0.001). BrWall_T2-MIS showed a moderate inverse correlation with PFTs in the whole cohort (*r* = −0.54, *r* = −0.44 for FEV1(%pred) and FEV1/FVC respectively, *p* ≤ 0.01) and in the severe asthma group (*r* = −0.53, *r* = −0.44 for FEV1(%pred) and FEV1/FVC respectively, *p* ≤ 0.01). Eos was moderately correlated with BrWall_T2-MIS in severe asthma group (*r* = 0.52, *p* = 0.047). Reproducibility was almost perfect with ICC = 0.99 and mean difference in Bland-Altman analysis of −0.15 [95% CI = −0.48–0.16].

**Conclusion:**

Quantification of bronchial wall T2w signal intensity appears to be able to differentiate severe from non-severe asthma and correlates with obstructive PFTs’ parameters and inflammatory markers in severe asthma.

**Critical relevance statement:**

The development of non-ionizing imaging biomarkers could play an essential role in the management of patients with severe asthma in the current era of biological therapies.

**Key Points:**

- Severe asthma exhibits severe exacerbations with a high burden on healthcare systems.
- T2w bronchial wall signal intensity is related to inflammatory biomarker in severe asthma.
- T2w MRI may represent a non-invasive tool to follow up severe asthma patients.

**Graphical Abstract:**

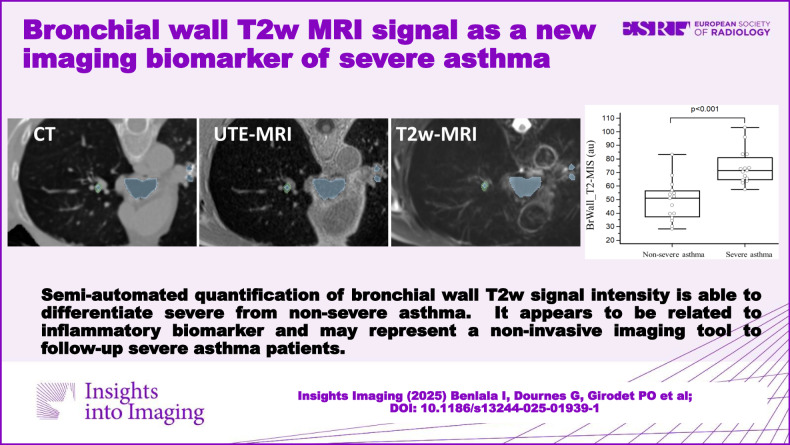

## Introduction

Severe asthma represents 3 to 5% of all asthmatic patients but causes around 60% of the disease costs [1]. Patients with severe asthma are known to be prone to severe exacerbations with a need for hospital admission, in addition to biologic medications, both leading to an increase in the economic burden of asthma management in the healthcare system [1]. Compared to non-severe asthmatics, severe asthmatic patients are characterized by increased levels of both sputum [2] and blood eosinophils [3]. However, 1.6 to 19.6% of severe asthmatic patients belong to the non-eosinophil and non-allergic endotype [4, 5]. Severe asthmatic patients are also characterized by different patterns of bronchial remodeling [6–8]. Both bronchial inflammation and remodeling can increase bronchial thickness [9].

Computed tomography (CT) is considered the reference technique to assess, in a non-invasive fashion, bronchial thickness in asthma. Indeed, bronchial wall measurements have been extensively assessed in asthma using CT and shown to be a reliable parameter to assess asthma severity [9–15]. Semi-automatic 2D and 3D techniques have been developed to assess bronchial dimensions [16, 17]. Thus, bronchial wall thickness assessed using CT was found to be associated with bronchial remodeling [9, 15] and with airflow obstruction assessed by lung function testing [10–13]. However, CT is an ionizing imaging modality, which limits its use in frequent long-term follow-up. In addition, specific bronchial inflammatory changes seem to remain invisible on CT, difficult to distinguish from bronchial remodeling [9]. By contrast, magnetic resonance imaging (MRI) is a non-ionizing 3D imaging technique recently becoming, with the development of ultra-short echo time (UTE) sequences, an alternative to CT in lung imaging [18–20]. A previous study demonstrated that UTE-MRI is an accurate and reliable radiation-free method to assess bronchial wall dimensions in asthma, able to differentiate patients with severe asthma from those with non-severe asthma [21]. Furthermore, T2-weighted (T2w) MRI was found to be useful in the assessment of airway inflammation [22, 23], and its response to treatment, both in cystic fibrosis [23, 24].

Thus, the primary objective of this study was to compare airways’ T2w mean signal intensity between patients with severe asthma and those with mild or moderate asthma. Secondary objectives were to evaluate relationships of the bronchial T2w mean signal intensity with pulmonary function tests (PFTs), fractional exhaled nitric oxide (FeNO), blood eosinophils count (Eos) and bronchial wall measurements on CT.

## Materials and methods

### Study population

Asthmatic patients, according to the “Global Strategy for Asthma Management and Prevention (GINA)” definition, aged more than 18 years were eligible for enrollment. Non-inclusion criteria were: (1) recent asthma exacerbation (less than 4 weeks from the study inclusion); (2) history of chronic obstructive pulmonary disease, lung fibrosis, pulmonary hypertension, lung cancer or cystic fibrosis; (3) pregnancy or breastfeeding woman and (4) MRI contraindications. Patients were categorized as either non-severe or severe asthma according to the ATS/ERS task force [25].

### Study design

This is an ancillary study from a prospective single-center study that was performed between May 2017 and June 2018 [21]. The local ethics committee approved the study, and all participants were required to give written informed consent (ClinicalTrials.gov number: NCT03089346).

Fifteen non-severe asthmatic patients were sex- and age-matched with 15 severe asthmatic patients. They had to undergo a chest CT scan and MRI-UTE on the same day with PFTs including the forced expiratory volume in 1 s (FEV1); forced volume capacity (FVC); forced expiratory flow at 25–75% of FVC (FEF25-75); FeNO and blood Eos within a maximum interval of 30 days. Asthma control questionnaire (ACQ) and asthma quality of life questionnaire (AQLQ) were reported for all patients [26, 27]. Scores on the ACQ range from 0 to 6, with lower scores indicating better asthma control. Scores on the AQLQ range from 1 to 7, with higher scores indicating better quality of life. In addition, we reported the number of exacerbations that occurred during the previous or the following 12 months from the MR examination. Exacerbation was defined according to the ERS/ATS task force as an asthma-related emergency department/hospital admission and/or primary care consultation and/or an acute oral corticosteroids (OCS) course of ≥ 3 days. Patients were categorized with either an asthma exacerbation rate (AER) ≥ 2 or an AER < 2.

We also performed additional comparison by subgrouping patients in: (1) High-Eos asthma (defined by blood eosinophil count ≥ 300/µL) vs Low-Eos asthma (defined by blood eosinophil count < 300/µL); (2) Chronic obstructive asthma (defined by FEV1 < 80% predicted) vs intermittent obstructive asthma (defined by FEV1 ≥ 80% predicted).

### MRI and CT examinations

Details of MRI and CT protocols are provided in the electronic supplementary methods. Briefly, two datasets were acquired: 3D-UTE morphological images using a prototypical sequence (Spiral Vibe for volume interpolated breath hold examination) [21] and a T2-weighted images (T2w) using periodically rotated overlapping parallel lines with enhanced reconstruction (PROPELLER). MR images were acquired at end normal expiration for both sequences using an automated navigator-triggered prospective synchronization.

Unenhanced chest CT images were acquired with a voxel size of 0.625 mm^3^ at end normal expiration (functional residual capacity (FRC)). Thus, both CT and MRI were acquired at the same lung volume (i.e., FRC).

### Image processing and bronchial wall area segmentation

First, since MRI is not a calibrated modality and to account for signal intensity variation between different patients, T2w images were normalized using the mean value and the standard deviation of the whole original image [28]. Briefly, images were normalized by centering them at their mean value with standard deviation of the whole image:

$$f\left(x\right)=\frac{\left({{{\rm{x}}}}-{\mu }_{x}\right)}{{\sigma }_{x}}+3{\sigma }_{x}$$

Then, CT images and T2w images were registered to UTE images (Supplementary Fig. E1) using the EVolution algorithm [29]. CT registered images were used to extract the bronchial tree using a previously validated homemade software (i.e., Neko) [17]. Briefly, it first reformatted registered CT images perpendicular to the bronchial axis. Second, it measured wall area (WA) and lumen area (LA) on these reformatted images using a Laplacian-of-Gaussian filter. Bronchial wall segmentation masks of a set of four bronchial paths at the third generation (i.e., right upper lobe, right lower lobe, left upper lobe and left lower lobe) were then extracted on axial images with two measurements of each path (Supplementary Fig. E2). Bronchus was excluded from analysis if proximal mucus plugging was present. Finally, since all the images were aligned on the same spatial domain, segmentation masks were applied on T2w images (Supplementary Fig. E3) allowing measuring the T2w bronchial wall area MRI signal. Misalignment was assessed visually and manual corrections were performed in case of inaccurate wall area segmentation.

### Bronchial wall area T2 mean signal intensity measurements (BrWall_T2-MIS)

**Fig. 1:**
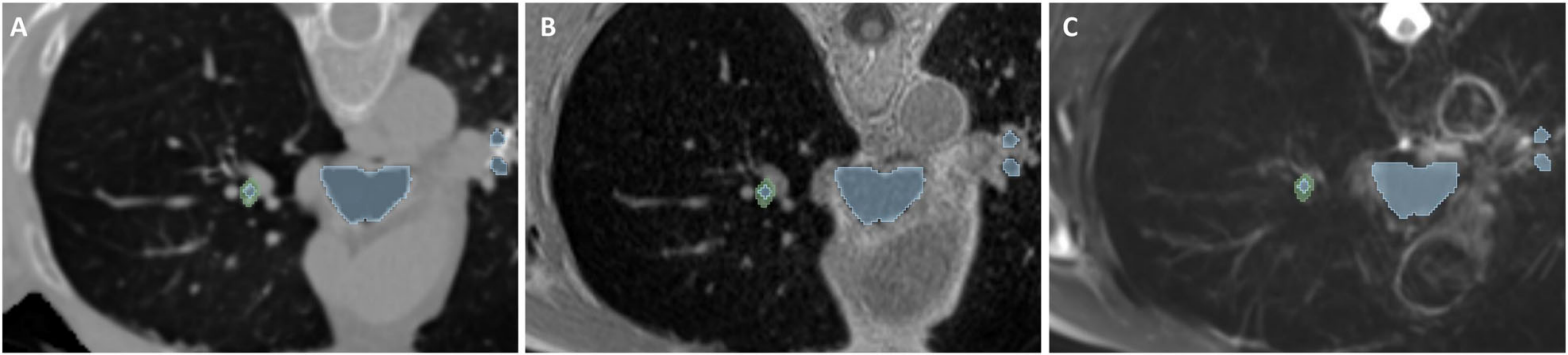
The bronchial wall segmentation mask of the right upper lobe bronchus, applied on the three modalities after elastic registration (perpendicular plane to the bronchus axis of (**A**) registered CT, (**B**) UTE MRI and (**C**) registered T2w MRI). Bronchial wall segmentation in green, airway lumen in blue. Visualization using 3D slicer

Wall area masks applied on T2w images allowed to measure automatically the mean signal intensity of the bronchial wall for each bronchial path (Fig. 1). The measurements were performed in random order and blinded from other participant’s data. The *BrWall_T2-MIS* measured over the four bronchial paths were averaged to get a single mean value per patient as the primary outcome measure. To assess inter-reader reproducibility of the measurement, IB and GD performed the measurements independently. The intra-reader reproducibility was also assessed using IB’s second measurements performed about a month apart from the first evaluation to prevent recall bias.

In addition, using the bronchial tree segmentation on CT images, the normalized wall area (WA%) was calculated on CT images as the percentage of WA divided by the sum of the LA and WA, as described previously [21]. We also grouped patients in four different groups using the quartiles of either WA% or *BrWall_T2-MIS* as a post hoc analysis.

### Statistical analyses

Statistical analyses were performed using MedCalc software (Version 20.216), by IB under the supervision of PB. Distribution normality was assessed using the Shapiro–Wilk test. Log-transformation was performed when the distribution normality was rejected. Data were expressed as mean ± standard deviation for continuous variables or geometric mean and standard deviation for log-transformed continuous variables and absolute number for categorical variables. Comparisons of quantitative and categorical variables were performed using Student’s *t*-tests and Chi-square or Fisher’s Exact tests, respectively. Comparison of the four groups based on quartiles was assessed using Anova with Tukey post hoc test. Sensitivity, specificity, and area under (AUC) the receiver operating characteristic curve (ROC) were also calculated after determining a cutoff value of BrWall_T2-MIS measurement using Youden index (i.e., sensitivity + specificity -1). We also performed a step-wise logistic regression with an AER in the next 12 months from the MRI examination ≥ 2 as the dependent variable with PFTs, FeNO, blood Eos and BrWall_T2-MIS as potential predictors.

Correlations were assessed using Pearson coefficients. Reproducibility was assessed by using Bland-Altman plot with mean difference and limits of agreement (LoA) and by using intraclass correlation coefficients (ICCs), with mixed model analysis and absolute agreement option. ICC values were classified as null (= 0), slight (> 0 and < 0.20), fair (≥ 0.20 and < 0.40), moderate (≥ 0.40 and < 0.60), good (≥ 0.60 and < 0.80), very good (≥ 0.80 and < 0.95), and almost perfect (≥ 0.95) [30]. A *p*-value < 0.05 was considered statistically significant.

## Results

### Study population

Thirty participants were included and categorized into two groups age- and sex-matched 15 non-severe and 15 severe asthmatic patients. In addition to age and sex, the two groups were similar in terms of body mass index (BMI), tobacco consumption and asthma duration (Table 1). Not surprisingly, the level of asthma control and the quality of life were worse despite higher dose of inhaled corticosteroids (ICS) and long-acting bronchodilators treatment in the severe asthma group. Moreover, the numbers of exacerbations within the 12 months before and after the MRI were both higher in patients with severe asthma whereas FEV1 and FEF_25-75%_ were significantly lower in the severe asthma group (Table 1). By contrast, both FeNO and blood eosinophil counts were not statistically different between the two groups.

**Table 1:** Asthma patients’ characteristics

		Non-severe	Severe	*p*-value
*n*		15	15	
Age	Years	50 ± 15	51 ± 16	0.89
Sex ratio	Men/women	1/14	1/14	1.00
BMI*	kg.m^−2^	26 ± 1.3	26 ± 1.2	0.68
Tobacco	Never smoker	12	11	1.00
	Former smoker	3	4	1.00
	Pack year (no)^£^	0 (0–0.7)	0 (0–0)	0.71
Questionnaire	ACQ	0.7 ± 0.9	2.3 ± 1.5	< 0.01
	AQLQ	6.2 ± 0.7	4.1 ± 1.1	< 0.01
Asthma duration	Years	31 ± 15	21 ± 14	0.07
Exacerbation	Number in the previous 12 months^£^	0 (0–0)	3 (2–5)	< 0.01
	Number in the following 12 months^£^	0 (0–0)	2 (1–5)	< 0.01
PFT	FEV_1_ (% pred)	101 ± 18	80 ± 26	0.01
	FVC (% pred)	108 ± 15	94 ± 21	0.06
	FEF_25-75_ (% pred)	83 ± 34	51 ± 36	0.01
	PEF (L/min)	418 ± 106	314 ± 116	0.01
	FeNO (ppb)*	21 ± 1.5	21 ± 1.6	0.94
	Blood Eos (cells/µL)*	128 ± 2	165 ± 3	0.49
Treatment	ICS (µg/day)	716 ± 844	2166 ± 1128	< 0.01
	OCS (yes/no)	0/15	2/13	0.48
	LABA (yes/no)	7/8	14/1	0.01
	LAMA (yes/no)	0/15	8/7	< 0.01
	LTRA (yes/no)	1/14	9/6	< 0.01
	Biologic (yes/no)	0/15	5/10	0.09

Data are mean ± SD for continuous variables and absolute number for categorical variables

*BMI* body mass index, *ACQ* Asthma Control Questionnaire, *AQLQ* Asthma Quality of Life Questionnaire, *PFT* pulmonary function tests, *FEV1* forced expiratory volume in 1 s, *FVC* forced volume capacity, *FEF25-75* forced expiratory flow at 25–75% of FVC, *%pred* percentage of predicted value, *PEF* peak expiratory flow, *FeNO* fractional exhaled nitric oxide, *Eos* eosinophils count, *ICS* inhaled corticosteroids, *OCS* continuous oral corticosteroids, *LABA* long-acting beta-agonists, *LAMA* long-acting muscarinic antagonists, *LTRA* leukotriene receptor antagonists

* Log-transformed variables with geometric mean and SD after back transformation

^£^ Medians with 95% confidence interval

### Comparisons of the T2w bronchial wall area intensity signal

**Fig. 2:**
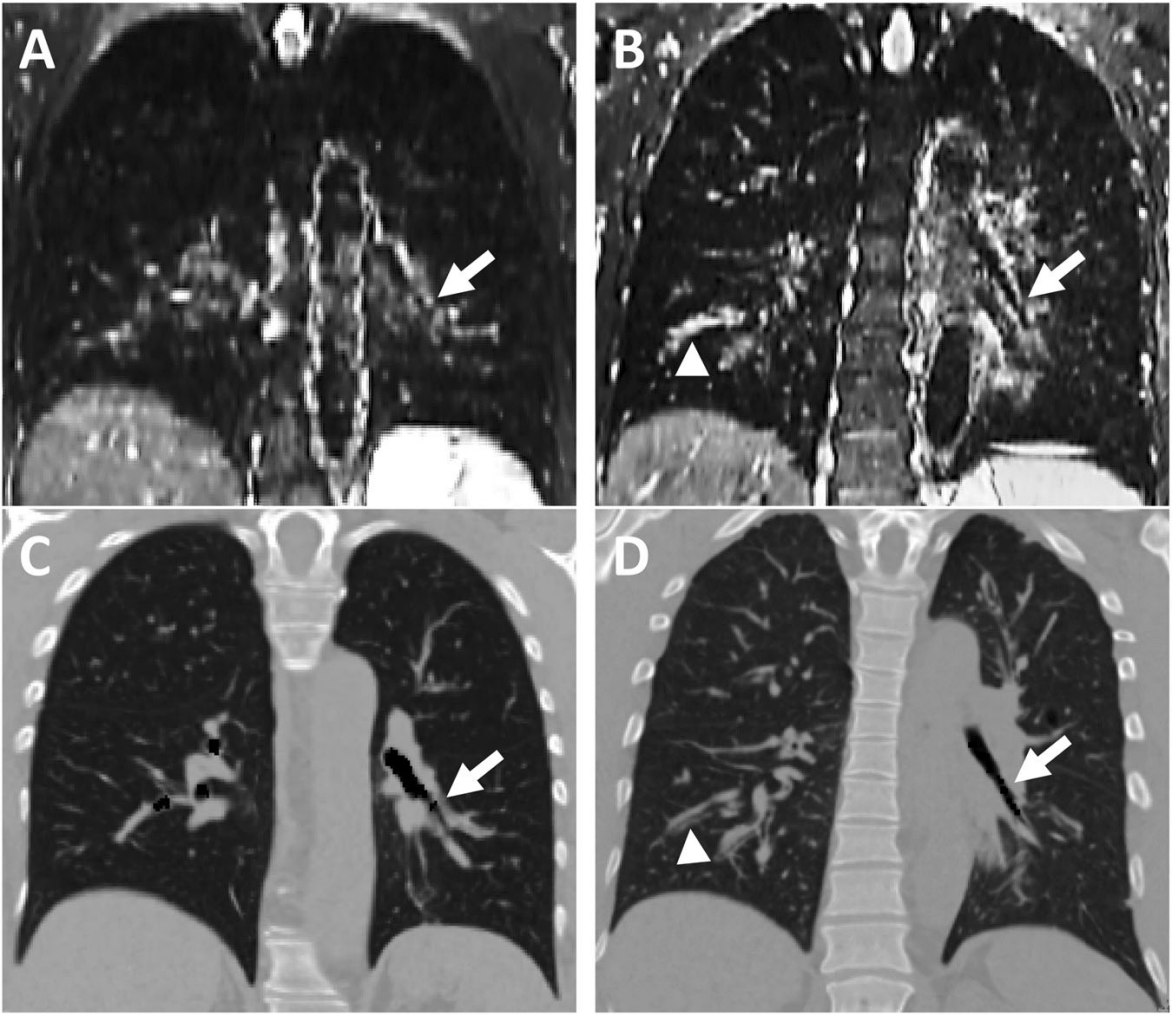
Coronal plane of registered T2w MRI (**A**, **B**) and registered CT (**C**, **D**) of a 48-year-old non-severe asthmatic female patient (left panels) and a 47-year-old severe asthmatic male patient (right panels). White arrows show the left lower lobe bronchus. Note a higher bronchial wall intensity signal on T2w images in severe asthmatic patient (**B**) in comparison to non-severe asthmatic patient (**A**) also indicated by the white arrowhead in sub segmental bronchus of the right lower lobe (**B**). BrWall_T2-MIS = 63 and 93 in non-severe and severe asthmatic patients, respectively

**Fig. 3:**
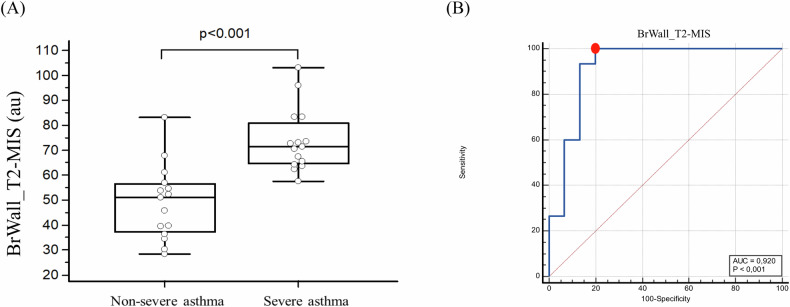
**A** Comparison of T2w bronchial wall mean signal measurements (BrWall_T2-MIS) between severe and non-severe asthma. Au means arbitrary unit. **B** Receiver operator characteristics curve of BrWall_T2-MIS measurements to distinguish between severe asthmatic and non-severe asthmatic patients. The red point represents the best sum of sensitivity and specificity

In the severe asthma group, BrWall_T2-MIS was significantly higher than in the non-severe asthma patients (74 ± 12 vs 49 ± 14; respectively *p* < 0.001) (Figs. 2,  3A). A cutoff value of 57 of BrWall_T2-MIS showed the best sum of sensitivity and specificity. Indeed, the sensitivity was 100% (95% CI: 78–100%) and the specificity was 80% (95% CI: 52–96%) with an AUC of 0.92 (95% CI: 0.76–0.99) (*p* < 0.001) to discriminate severe from non-severe asthmatic patients (Fig. 3B). In addition, among PFTs, FeNO, blood Eos and MRI, BrWall_T2-MIS was the sole predictive variable included in the step-wise logistic regression model, with an AER in the following 12 months ≥ 2 as the dependent variable, showing an odds ratio of 1.11 [95% CI: 1.02–1.20].

**Fig. 4:**
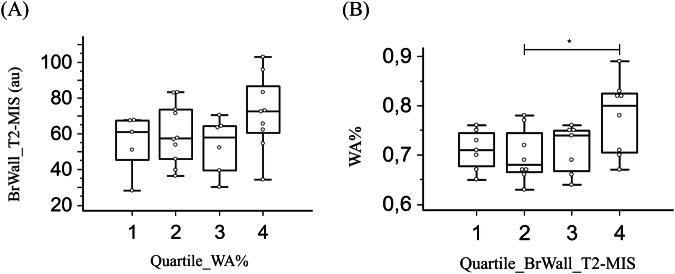
**A** Comparison of BrWall_T2-MIS among the four groups determined using quartile of WA%. **B** Comparison of WA% among the four groups determined using quartile of BrWall_T2-MIS. (*) represents a significant difference of Tukey post hoc test between the 4th and the 2nd quartile (*p* = 0.046)

We then analyzed the value of BrWall_T2-MIS compared to the normalized wall area (WA%) assessed previously at CT scan [21]. We did not find any significant difference in BrWall_T2-MIS among the patients grouped according to the quartiles of WA% (*p* = 0.21, Fig. 4A). By contrast, there was a small significant difference in WA% among the patients grouped according to the quartiles of BrWall_T2-MIS (*p* = 0.046, Fig. 4B). In addition, the ROC curve analysis of WA% to distinguish severe from non-severe asthma patients showed a trend to a lower AUC using an optimal cutoff of WA% of 73% (AUC = 0.74; 95% CI: 0.54–0.88) (*p* = 0.01, Supplementary Fig. E4). However, the pairwise comparison of ROC curves was not statistically different (*p* = 0.08).

We also compared BrWall_T2-MIS between other subgroups of asthma, whose characteristics were presented in Tables E1 & E2. There was a trend toward a difference in BrWall_T2-MIS between High-Eos (*n* = 7) and Low-Eos (*n* = 23) asthma groups (73 ± 22 vs 58 ± 16, respectively, *p* = 0.06). Of note, in High-Eos group, 5 of 7 patients exhibited severe asthma and in Low-Eos group, 10 of 23 patients were categorized as severe asthma patients (*p* = 0.20). However, we found that BrWall_T2-MIS was significantly higher in chronic obstructive (*n* = 9) than in intermittent obstructive asthma group (*n* = 21) (77 ± 16 vs 54 ± 15, respectively, *p* < 0.001). In chronic obstructive asthma group, 8 patients of 9 were categorized as severe asthma patients and they were 7 of 14 in intermittent obstructive group (*p* < 0.01).

On the other hand, WA% CT did not show any significant difference between these subgroups (74 ± 4 vs 71 ± 6; *p* = 0.30).

### Correlations of T2w bronchial wall area intensity signal with other outcomes

In the whole cohort, BrWall_T2-MIS showed a moderate inverse correlation with PFTs obstructive parameters (*r* = −0.54, *r* = −0.44 with FEV1(%pred) and FEV1/FVC respectively, *p* ≤ 0.01). In the severe asthma group, T2w bronchial wall area intensity signal measurement showed similar correlations (*r* = −0.53, *r* = −0.44 for FEV1(%pred) and FEV1/FVC respectively, *p* ≤ 0.01) but not in the non-severe asthma group (*p* > 0.25) (Table 2). Significant moderate correlation between T2w bronchial wall area intensity signal measurement and blood Eos was only found in the severe asthma group (*r* = 0.52, *p* = 0.047). No significant correlation was found between BrWall_T2-MIS and FeNO neither in the whole cohort, nor in the two groups (Table 2). Of note, WA% CT did not show any significant correlation (*r* = 0.36, *p* = 0.17 in severe asthma group).

**Table 2:** Correlations of T2w bronchial wall mean signal (BrWall_T2-MIS) with pulmonary function tests, FeNO and Blood Eos

PFT’s	T2w bronchial wall mean signal (BrWall_T2-MIS)		
	Whole population (*n* = 30)	Severe asthma (*n* = 15)	Non-severe asthma (*n* = 15)
FEV_1_(%pred)	−0.54 **(0.001)**	−0.53 **(0.002)**	−0.31 (0.25)
FVC (%pred)	−0.46 **(0.01)**	−0.47 (0.07)	−0.21 (0.43)
FEV_1_/FVC	−0.44 **(0.01)**	−0.44 **(0.01)**	−0.17 (0.54)
FEF_25-75_ (%pred)	−0.49 **(0.006)**	−0.38 (0.16)*	−0.22 (0.41)
PF (L/min)	−0.37 **(0.042)**	−0.16 (0.55)	−0.09 (0.75)
Blood Eos (cells/µL)	0.36 (0.051)*	0.52 (0.047)*	0.08 (0.76)
FeNO (ppb)	0.06 (0.76)*	0.49 (0.11)*	−0.18 (0.52)

Bold values indicate statistical significance *p* < 0.05.

Data are Pearson correlation coefficients with *p*-value in parentheses

*FEV1* forced expiratory volume in 1 s, *FVC* forced volume capacity, *FEF25-75* forced expiratory flow at 25–75% of FVC, *%pred* percentage of predicted value, *PEF* peak expiratory flow, *FeNO* fractional exhaled nitric oxide, *Eos* eosinophils count

* Log-transformed variables

### Reproducibility of the BrWall_T2-MIS measurements

The intra and inter-observer reproducibility was almost perfect with ICC = 0.99 [95% CI = 0.99; 0.99] and mean difference in Bland-Altman analysis of 0.04 [95% CI = −0.32; 0.42] with LoA [−1.96; 2.04] for intra-observer measurements and −0.15 [95% CI = −0.48; 0.16] with LoA [−1.84; 1.53], for inter-observer measurements (Supplementary Figs. E5 and E6).

## Discussion

Taken together, we demonstrated that BrWall_T2-MIS, measured using proton MRI without any contrast agent or hyperpolarized gas, could be considered a new imaging biomarker of severe asthma. Indeed, BrWall_T2-MIS is increased in severe asthma compared to non-severe asthma and is able to predict the presence of severe asthma among asthmatic patients with maximal sensitivity and high specificity. We also found that BrWall_T2-MIS is the sole predictor of a high rate of exacerbations (i.e., AER ≥ 2) among other PFTs and biological variables. Moreover, it is highly reproducible and seems to be partly independent of the wall area but related to both functional obstruction and blood eosinophils.

This pilot study demonstrated that T2w MRI signal within the bronchial wall area can be measured in patients with asthma using semi-automatic tools. Our pre-processing method allows us to take benefit of the high resolution of CT by using a robust multimodal registration method to segment bronchial wall area on T2-MRI images semi-automatically and in a reliable way with an almost perfect inter-observer agreement. Indeed, our semi-automatic technique relying on image registration and well-validated bronchial wall segmentation tools allows us to bypass the need for subtle visual analysis of MR images.

Our results suggest that bronchial T2 signal is related to both asthma severity and inflammatory mechanisms. Indeed, we found a positive correlation between BrWall_T2-MIS and blood eosinophils, a well-known biomarker of airway inflammation, in severe asthmatic patients [31]. The absence of correlation between BrWall_T2-MIS and FeNO has to consider the high variability of FeNO on the one hand, and, on the other hand, the poor indirect relationships between both blood and sputum eosinophils with FeNO [32, 33]. There was only a trend of a difference in BrWall-T2-MIS between High-Eos and Low-Eos subgroups, but the subpopulations’ sizes were not powered enough. On the other hand, WA% CT did not show any significant difference between these subgroups and no significant correlation with blood Eos. However, patients with the highest wall area did not have increased values of BrWall-T2-MIS, suggesting that bronchial wall area and bronchial T2w signal were two different biomarkers and might be related to bronchial remodeling and bronchial inflammation respectively. In addition, the ROC curve analysis of BrWall_T2-MIS to predict severe asthma showed a trend to a higher AUC compared to that of WA% with 100% sensitivity. This suggests that severe asthma heterogeneity can be captured using T2-MRI, whereas WA% reflects the global bronchial wall thickening without distinguishing between inflammation and remodeling that can be present in non-severe asthma as previously reported by our team [8]. Indeed, bronchial wall thickness evaluated on CT images encompasses both bronchial remodeling and inflammation. To our knowledge, CT techniques capable of distinguishing remodeling from inflammation have not been reported yet. CT bronchial wall attenuation in asthma was evaluated demonstrating a high correlation with obstructive PFT parameters [34], with one study reporting positive correlation with Non-degranulated mast cell count in only nine patients [35]. Nonetheless, the need for histology on bronchial biopsies may enlighten the capabilities of CT and MRI to distinguish between bronchial remodeling and inflammation.

Severe asthma is also characterized by greater obstruction [36]. The present study shows significant correlations between BrWall-T2-MIS and both FEV1 and FEV1/FVC particularly in the severe asthma group. We also found a higher BrWall-T2-MIS in the subgroup of patients with persistent airflow limitation compared to that with intermittent airflow limitation. Airflow limitation in asthma is thought to be related to inflammatory mechanisms, and it has been shown that persistent airflow limitation is associated with a high level of blood Eos [37].

Vogel-Claussen et al previously showed the feasibility of quantifying pulmonary inflammation using Turbo-Inversion Recovery Magnitude MRI sequence after segmental allergen challenge regardless of the disease severity [38]. This study is the first to quantify pulmonary inflammation in severe asthma in comparison to mild or moderate asthma using proton MRI without any contrast agent. Indeed, other previous studies evaluating MRI in severe asthma were performed using contrast agent inhalation (Xe^129^) [39, 40] to monitor the response to bronchial thermoplasty, which represents the ultimate alternative in the treatment of severe asthma.

In our study, severe asthmatic patients used more drug therapy, including corticosteroids, which may have a potential impact on T2w signal of the bronchial wall. However, our findings seem to suggest that bronchial wall T2w signal is a robust biomarker and reflects the underlying chronic inflammation distinguishing between severe and non-severe asthma apart from any exacerbation. In addition, severe asthma patients are generally under triple therapy including ICS and the fact that our biomarker is able to distinguish these patients makes a more reliable tool in the assessment of severe asthma.

Biotherapy represents a major asset in the management of patients with severe asthma. However, in order to assess the efficacy of such therapies, experts recommend extending treatment for several months. Moreover, the high cost of these new therapies increases the economic burden of severe asthma on the healthcare system. Nevertheless, the selection of patients who can benefit from these therapies is well established, and response predictors are available to help clinicians decide whether or not to initiate biologic treatment [41]. However, the lack of a morphological tool to objectively monitor the response to these treatments prevents clinicians from modifying earlier their approach and management. The tool developed in this study could be an additional asset in clinicians’ toolbox for managing severe asthma in a non-invasive, radiation-free way. Indeed, our study showed that BrWall_T2-MIS could be considered an independent predictor of high AER in the following 12 months.

Our study has some limitations. First, it is an ancillary study with a post hoc design. Nevertheless, in the original study, we were particularly rigorous in recruiting age- and sex-matched groups of asthma patients of distinct severity, and we collected all the necessary data useful for this ancillary study. Second, this was a pilot study with a small number of participants as a proof of concept using novel imaging techniques to demonstrate the feasibility and the utility of T2w MRI in severe asthma. Larger multicenter studies are still needed to validate the applicability and clinical significance of this method. Moreover, the cutoff value for the mean T2 bronchial wall area signal would have to be validated in future multicenter studies. Third, the spatial resolution of T2w images remains well below that of CT scans. However, the T2w Blade sequence has been found to exhibit less artifacts and better image quality in comparison to standard T2w sequences [42]. Moreover, we have achieved very good reproducibility with our method, which takes advantage of the morphological superiority of CT scans and registration tools to segment the bronchial wall area as accurately as possible on MRI T2w images. Fourth, it would have been necessary to perform histology on bronchial biopsies to clearly differentiate bronchial inflammation from remodeling, as we did previously in a 3D-CT scan study [9].

In conclusion, we demonstrated that the quantification of bronchial wall area MRI T2w signal is feasible in asthmatic patients and that this new biomarker is a valid tool to distinguish severe from non-severe asthma. Thus, the use of MRI as an imaging biomarker of inflammation in asthma could play an essential role in the management of severe asthma, especially in the era of biotherapies.

## Supplementary information

figureSuppl1R1

figureSuppl2R1

figureSuppl3R1

figureSuppl4R1

figureSuppl5R1

figureSuppl6R1

Supplementary MaterialR1

## Data Availability

The data are available upon reasonable request from the authors.

## Abbreviations

AUC: Area under the curve
BrWall_T2-MIS: Bronchial wall T2 mean intensity signal
CI: Confidence interval
Eos: Blood eosinophils count
FeNO: Fractional exhaled nitric oxide
ICC: Intraclass correlation coefficient
ICS: Inhaled corticosteroid
LoA: Limits of agreement
PFT: Pulmonary function tests
ROC: Receiver operator characteristics curve
UTE: Ultra-short echo time
