# Supplementary figures and images for: Bronchial wall T2w MRI signal as a new imaging biomarker of severe asthma
Source: Insights Imaging. 2025 Mar 25;16:71. doi: 10.1186/s13244-025-01939-1 (PMC11937477; doi:10.1186/s13244-025-01939-1)

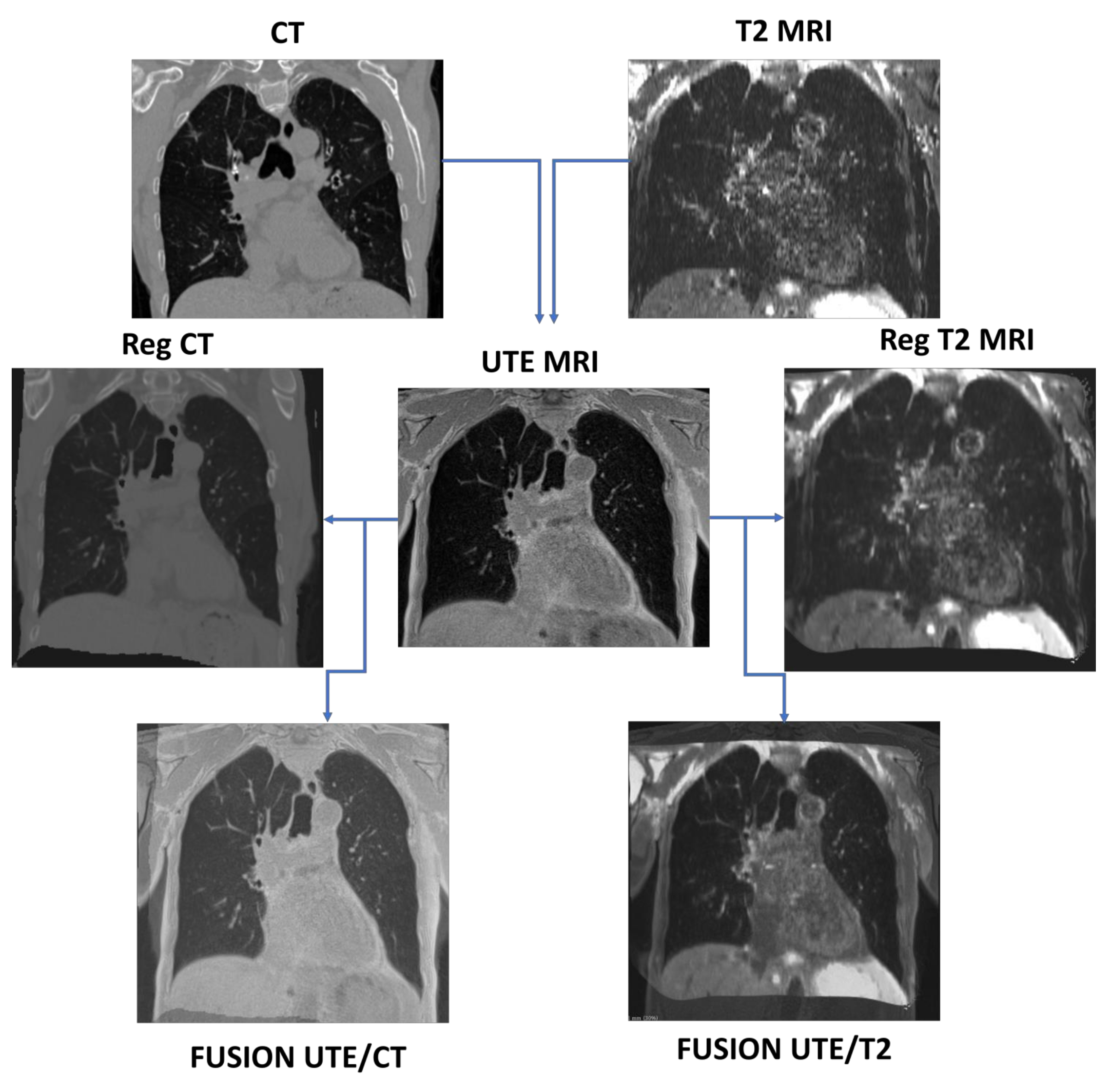

Supplement: Supplementary file 1 — figureSuppl1R1 [file 13244_2025_1939_MOESM1_ESM.tiff]

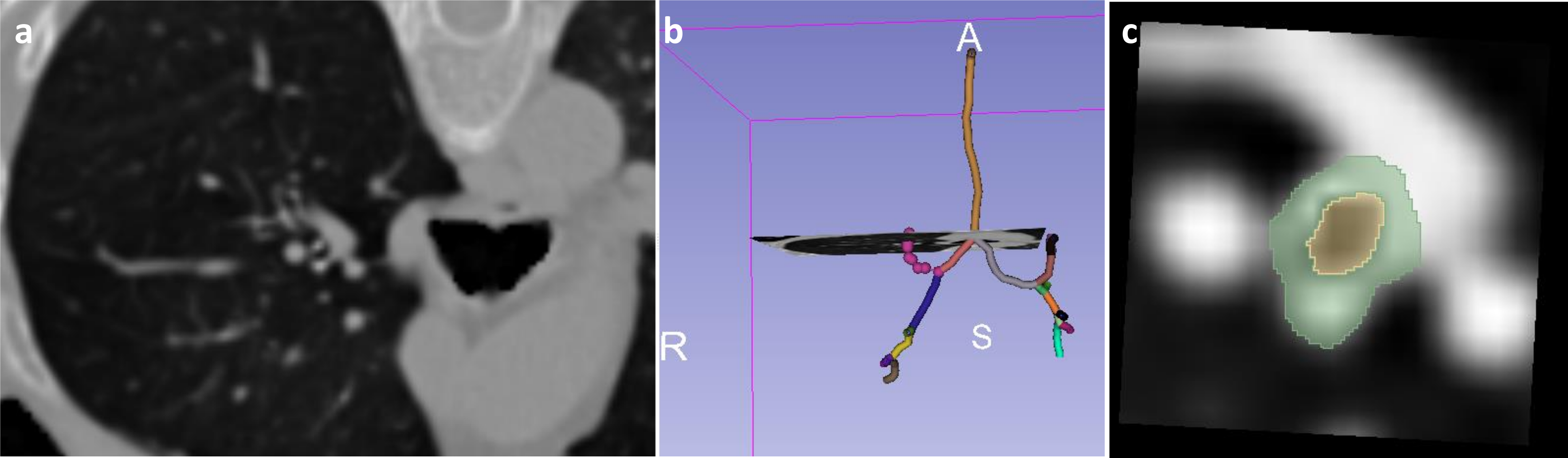

Supplement: Supplementary file 2 — figureSuppl2R1 [file 13244_2025_1939_MOESM2_ESM.tiff]

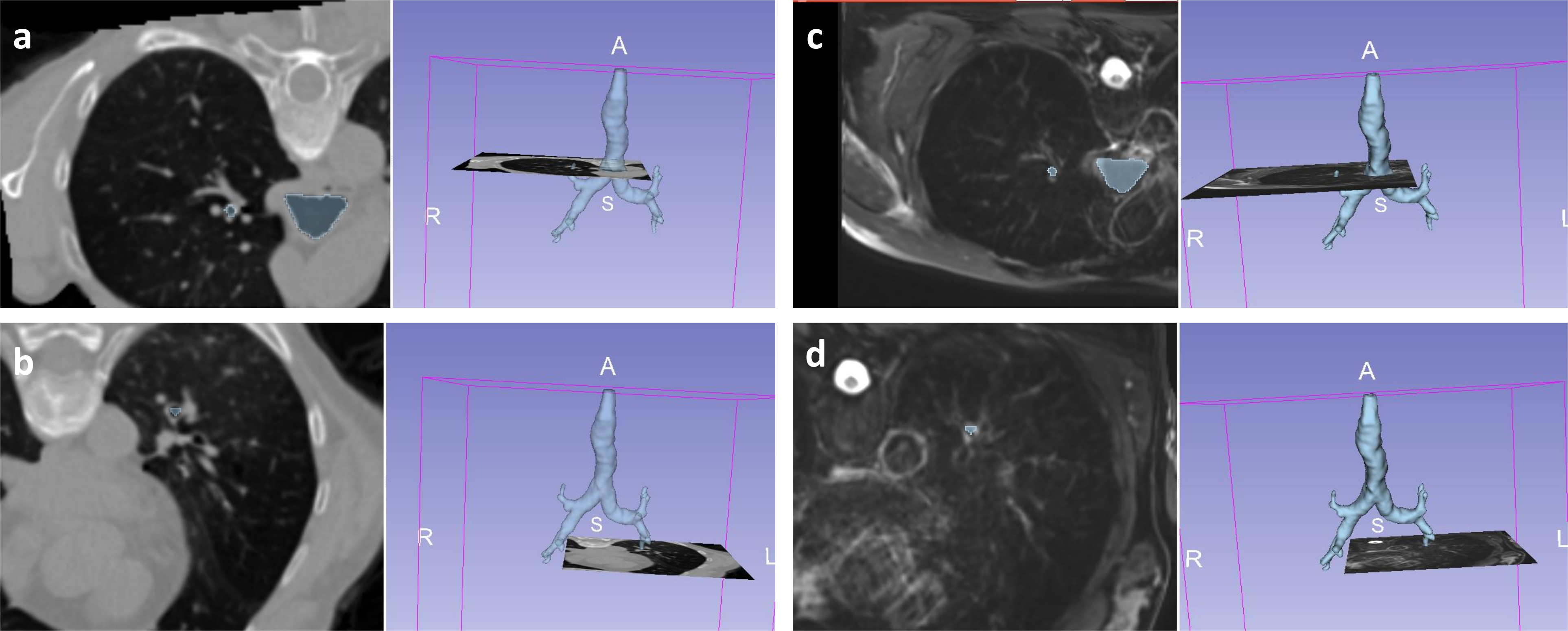

Supplement: Supplementary file 3 — figureSuppl3R1 [file 13244_2025_1939_MOESM3_ESM.tiff]

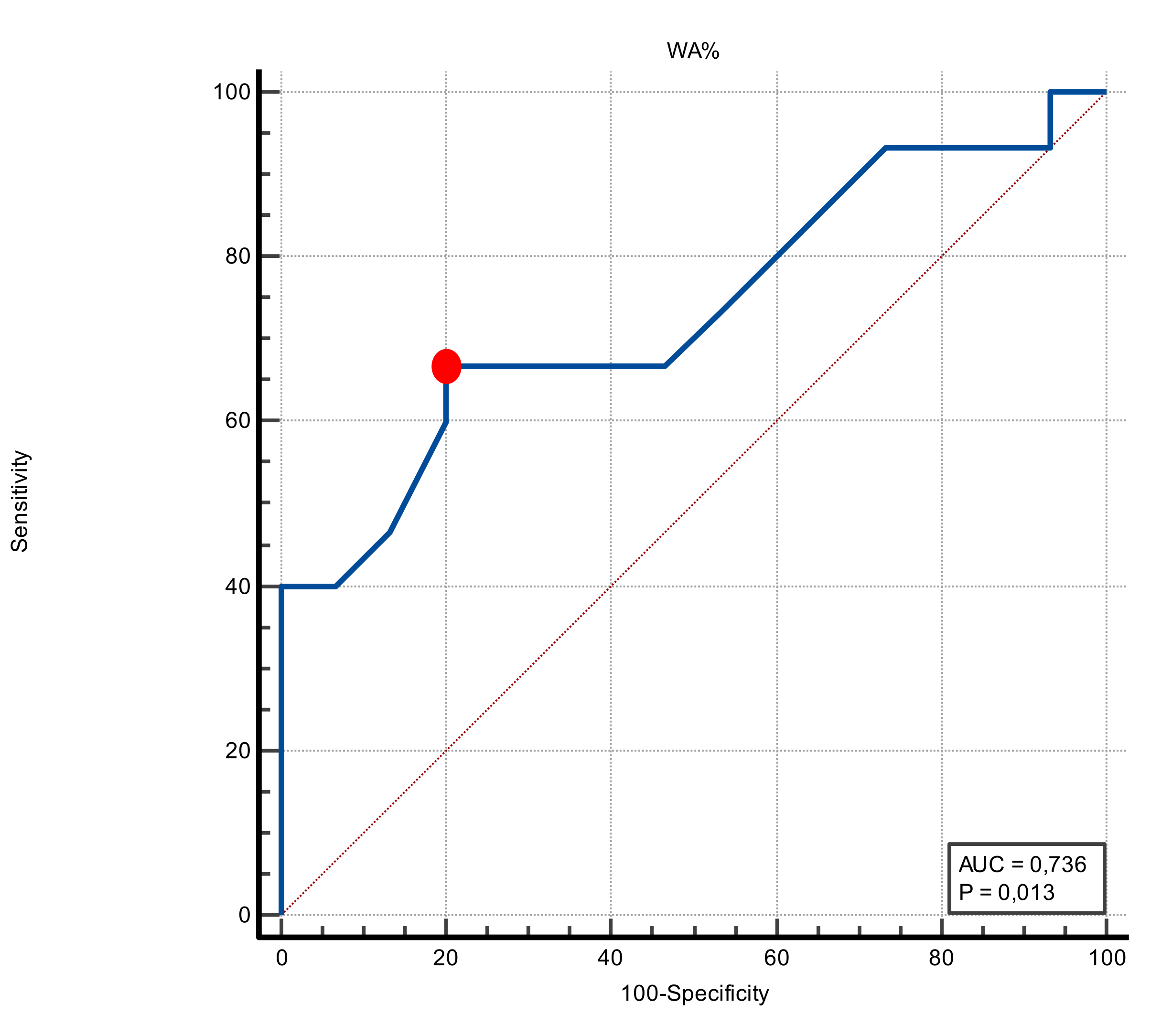

Supplement: Supplementary file 4 — figureSuppl4R1 [file 13244_2025_1939_MOESM4_ESM.tiff]

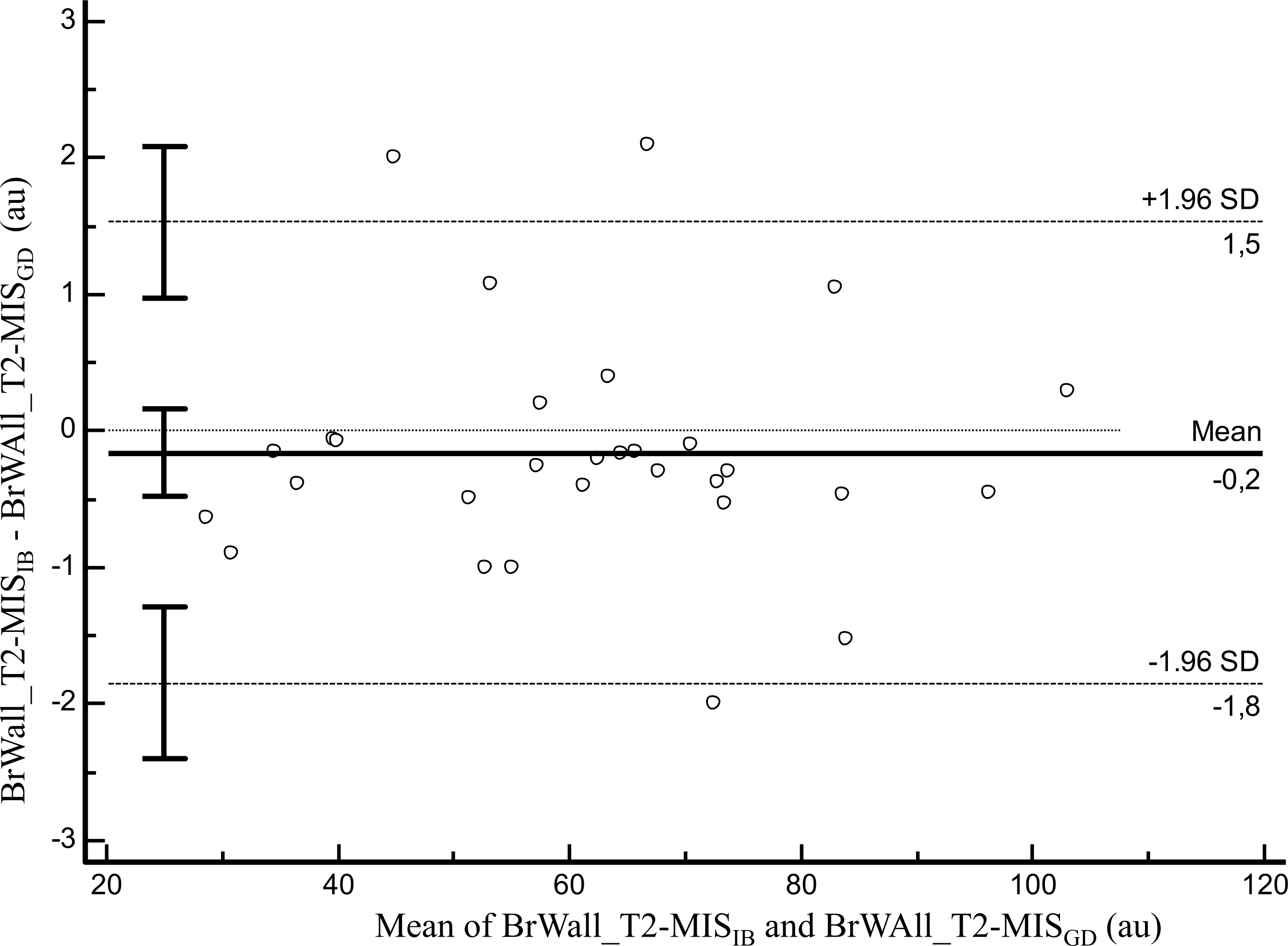

Supplement: Supplementary file 5 — figureSuppl5R1 [file 13244_2025_1939_MOESM5_ESM.tiff]

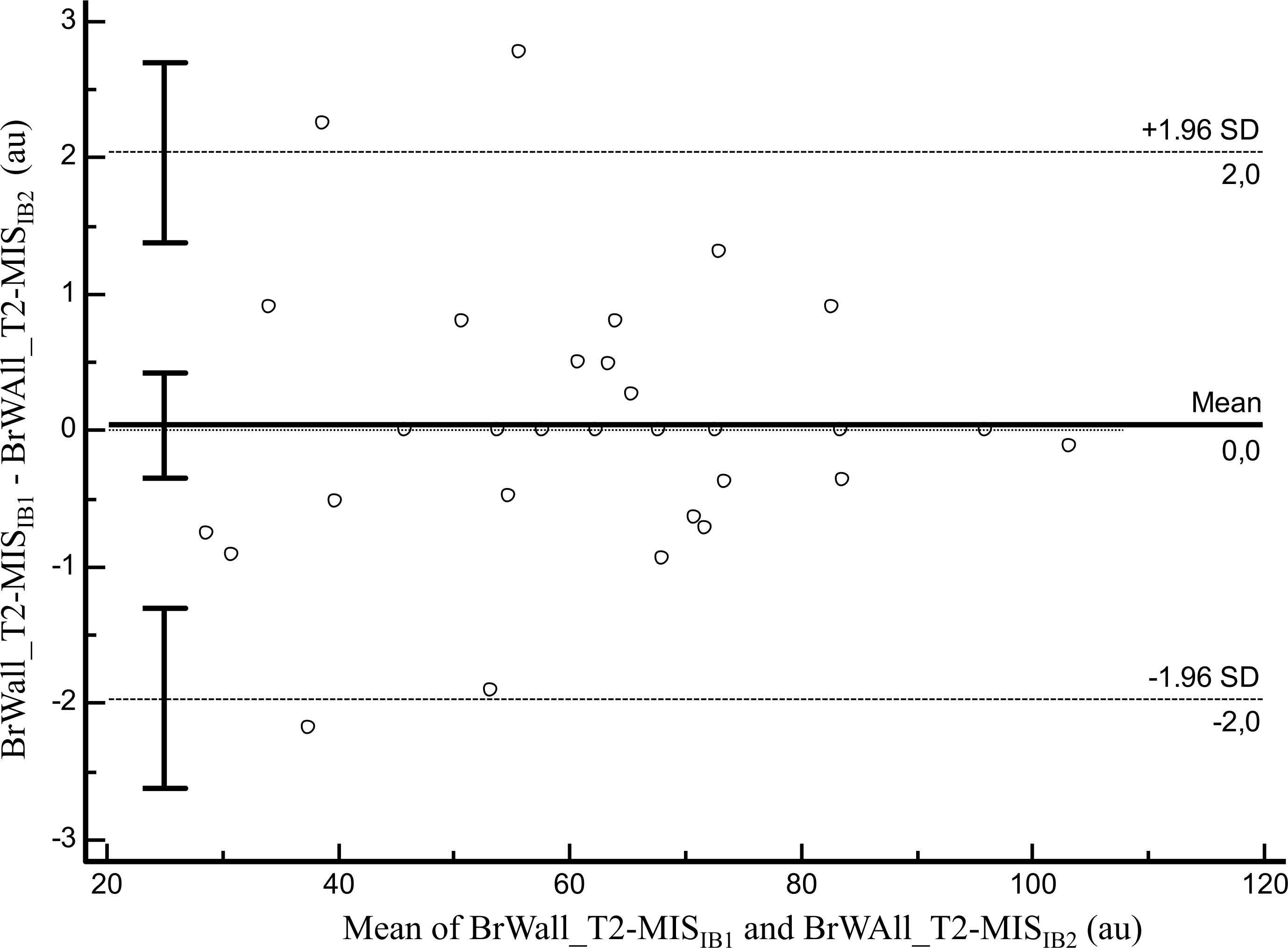

Supplement: Supplementary file 6 — figureSuppl6R1 [file 13244_2025_1939_MOESM6_ESM.tiff]
