# Supplementary material for: Bronchial wall T2w MRI signal as a new imaging biomarker of severe asthma
Source: Insights Imaging. 2025 Mar 25;16:71. doi: 10.1186/s13244-025-01939-1 (PMC11937477; doi:10.1186/s13244-025-01939-1)

Supplementary Methods

MRI was performed with a 1.5 Tesla system (MAGNETOM AVANTO; Siemens Healthcare, Erlangen, Germany). For the study purpose, two datasets were acquired. High-resolution morphological images of the lung were acquired in coronal plane using a prototypical spiral volume interpolated breath hold examination (VIBE) (3D-UTE) sequence (1). Respiratory synchronization at end normal expiration was allowed by an automated navigator-triggered prospective synchronization (2). Acquisition parameters were as follows: repetition time (TR) / echo time (TE) / flip angle = 4.1/0.07msec/5; coronal native acquisition plane; voxel size = $1mm^3$; acquisition time 6–7 minutes. A T2-weighted sequence (T2w) was obtained in axial plane by using periodically rotated overlapping parallel lines with enhanced reconstruction (PROPELLER), and more specifically, the BLADE method, which is a PROPELLER-equivalent implementation of the Siemens Medical System. T2-BLADE was acquired in free-breathing with a respiratory navigator placed at the level of the right diaphragm at end-normal expiration with the following parameters: TR/TE/flip angle = 2000/27msec/150; averages = 1; voxel size = $1.4 \times 1.4 \times 3$ mm^3; acquisition time 4–8 minutes. Unenhanced chest CT images were acquired with a 64-slice multidetector CT scanner (Somatom Definition; Siemens Healthcare, Erlangen, Germany) with a voxel size of 0.625 mm^3. Patients were instructed, before and during the procedure, to hold their breath at end normal expiration (functional residual capacity). Images were reconstructed using standard kernel (B30f). Thus, both CT and MRI were acquired at the same lung volume (i.e., functional residual capacity).

Supplementary references

1. Benlala I, Dournes G, Girodet P-O, Benkert T, Laurent F, Berger P. Evaluation of bronchial wall thickness in asthma using magnetic resonance imaging. Eur Respir J. 2022;59(1):2100329. doi: 10.1183/13993003.00329-2021.

2. Dournes G, Yazbek J, Benhassen W, et al. 3D ultrashort echo time MRI of the lung using stack-of-spirals and spherical k-Space coverages: Evaluation in healthy volunteers and parenchymal diseases. J Magn Reson Imaging JMRI. 2018;48(6):1489–1497. doi: 10.1002/jmri.26212.

Supplementary Tables

Supplementary Table E1. Patients' characteristics

		Low-Eos	High-Eos	p-value
**n**		23	7	
**Age**	Years	48 ± 16	47 ± 15	0.86
**Sex ratio**	Men/Women	0 / 23	2 / 5	0.048
**BMI***	kg.m^{-2}	26 ± 1.2	25 ± 1.2	0.58
**Tobacco**	Never smoker	17	6	0.52
	Former smoker	6	1	0.52
	pack year (no)	0 (0-0)	0 (0-4)	0.75
**Questionnaires**	ACQ	1.4 ± 1.4	1.7 ± 1.6	0.56
	AQLQ	5.2 ± 1.5	5 ± 1.2	0.70
**Asthma duration**	Years	28 ± 15	18 ± 15	0.15
**Exacerbations**	Number in the previous 12 months	1 (0-3)	1 (0-8)	0.52
	Number in the following 12 months	0 (0-2)	2 (0-7)	0.10
**PFT**	FEV_1 (% pred)	95 ± 24	77 ± 22	0.10
	FVC (% pred)	103 ± 20	95 ± 16	0.36
	FEF_{25-75} (% pred)	73 ± 38	49 ± 34	0.14
	PEF (l/min)	362 ± 125	378 ± 118	0.76
	FeNO (ppb)*	19 ± 1.5	27 ± 1.6	0.06
	Blood Eos (cells/µl)*	97 ± 1.9	541 ± 1.9	<0.01
**Treatment**	ICS (µg/day)	714 ± 638	908 ± 717	0.63
	OCS (yes/no)	2/21	0/7	1
	LABA (yes/no)	16/7	5/2	0.92
	LAMA (yes/no)	6/17	2/5	0.89
	LTRA (yes/no)	5/18	5/2	0.01
	Biologic (yes/no)	3/20	2/5	0.34

Data are mean ± SD for continuous variables and absolute number for categorical variables. (*) log transformed variables with geometric mean and SD after back transformation. (£) medians with (95% confidence interval). Abbreviations: BMI=body mass index; ACQ=Asthma Control

Questionnaire; AQLQ=Asthma Quality of Life Questionnaire; PFT= pulmonary function tests; FEV1=forced expiratory volume in 1 second; FVC=forced volume capacity; FEF25-75=forced expiratory flow at 25%-75% of FVC; %pred=percentage of predicted value; PEF=peak expiratory flow; FeNO=fractional exhaled nitric oxide; Eos=eosinophils count; ICS=inhaled corticosteroids; OCS=continuous oral corticosteroids; LABA=long-acting beta agonists; LAMA=long acting muscarinic antagonists; LTRA=leukotriene receptor antagonists.

Supplementary Table E2. Patients' characteristics

		Intermittent obstructive	Chronic obstructive	p-value
**n**		21	9	
**Age**	Years	46 ± 1.5	43 ± 1.4	0.62
**Sex ratio**	Men/Women	1/ 20	1/ 8	0.51
**BMI***	kg.m^{-2}	26 ± 1.3	26 ± 1.2	0.68
**Tobacco**	Never smoker	16	7	0.92
	Former smoker	5	2	0.92
	pack year (no)	0 (0-0)	0 (0-0)	0.36
**Questionnaires**	ACQ	0.8 ± 1	2.9 ± 1.4	<0.01
	AQLQ	5.7 ± 1.2	4.0 ± 1.2	<0.01
**Asthma duration**	Years	26 ± 14	25 ± 18	0.83
**Exacerbation**	Number in the previous 12 months	1 (0-2)	3 (1-6)	0.08
	Number in the following 12 months	0 (0-0)	2 (1-6)	<0.01
**PFT**	FEV_1 (% pred)	103 ± 16	61 ± 11	<0.01
	FVC (% pred)	109 ± 14	81 ± 15	<0.01
	FEF_{25-75} (% pred)	84 ± 33	28 ± 8	<0.01
	FEV_1/FVC	81 ± 9	65 ± 6	<0.01
	PEF (l/min)	411 ± 98	261 ± 108	<0.01
	FeNO (ppb)*	19 ± 1.5	25 ± 1.6	0.18
	Blood Eos (cells/µl)*	113 ± 2	257 ± 4	0.03
**Treatment**	ICS (µg/day)	658 ± 694	944 ± 572	0.28
	OCS (yes/no)	2/19	0/9	1
	LABA (yes/no)	12/9	9/0	0.08
	LAMA (yes/no)	5/16	3/6	0.66
	LTRA (yes/no)	4/17	6/3	0.03
	Biologic (yes/no)	2/19	3/6	0.11

Data are mean ± SD for continuous variables and absolute number for categorical variables. (*) log transformed variables with geometric mean and SD after back transformation. (£) medians with (95% confidence interval). Abbreviations: BMI=body mass index; ACQ=Asthma Control Questionnaire; AQLQ=Asthma Quality of Life Questionnaire; PFT= pulmonary function tests; FEV1=forced expiratory volume

in 1 second; FVC=forced volume capacity; FEF25-75=forced expiratory flow at 25%-75% of FVC; %pred=percentage of predicted value; PEF=peak expiratory flow; FeNO=fractional exhaled nitric oxide; Eos=eosinophils count; ICS=inhaled corticosteroids; OCS=continuous oral corticosteroids; LABA=long-acting beta agonists; LAMA=long acting muscarinic antagonists; LTRA=leukotriene receptor antagonists.

Supplementary Figure captions

Supplementary Figure E1. Elastic registration of CT and T2w MRI images on ultra-short echo (UTE) MRI images.

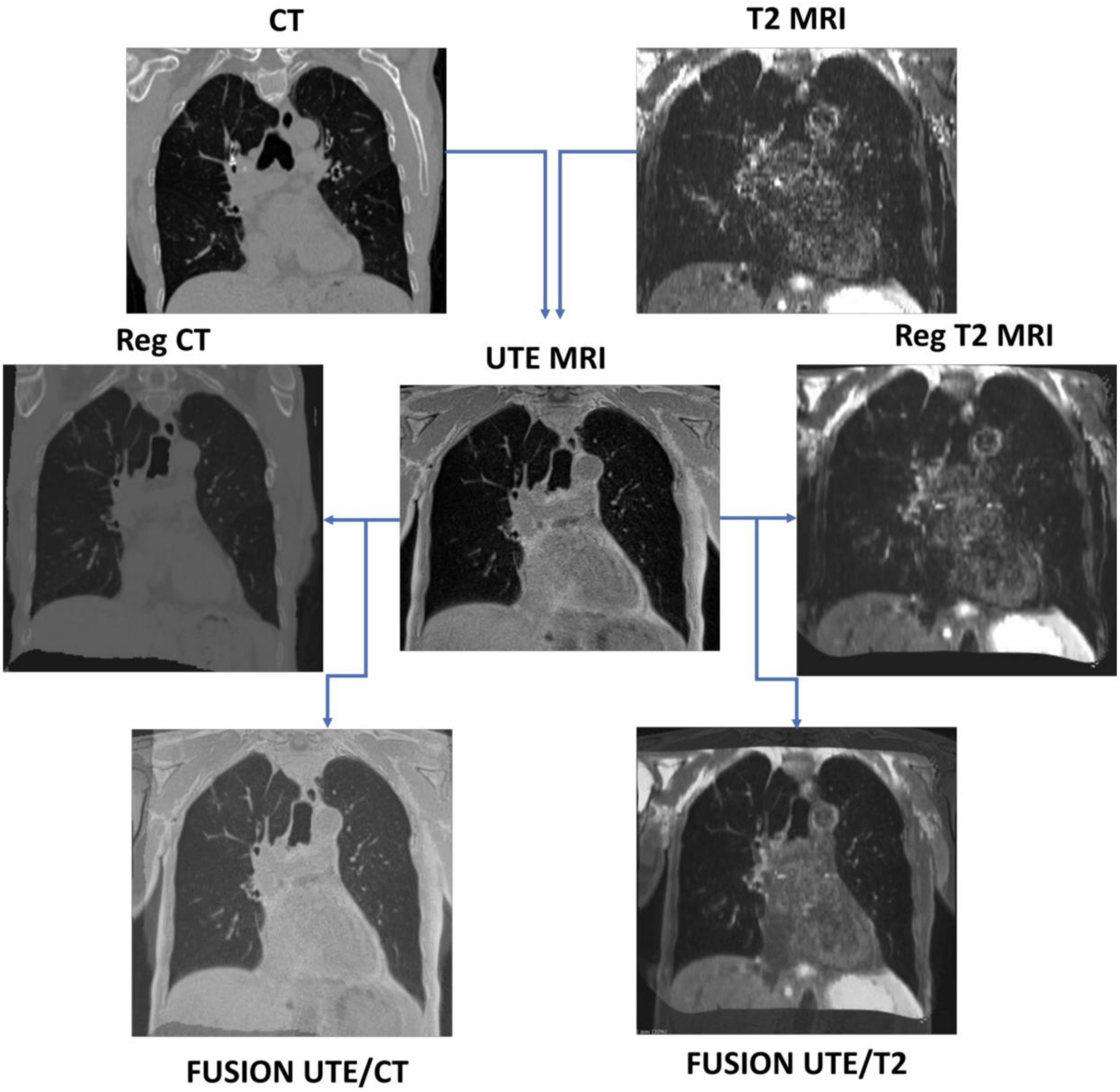

Supplementary Figure E2. Bronchial tree automatic extraction and semi-automatic segmentation of bronchial wall on CT images. (a) Axial CT image of severe asthma patient centred on right upper lobe bronchus (RB1). (b) Automatic bronchial tree extraction and skeletonization. (c) Bronchial wall area (WA) segmentation in green (bronchial lumen area in yellow). Visualisation using 3D slicer.

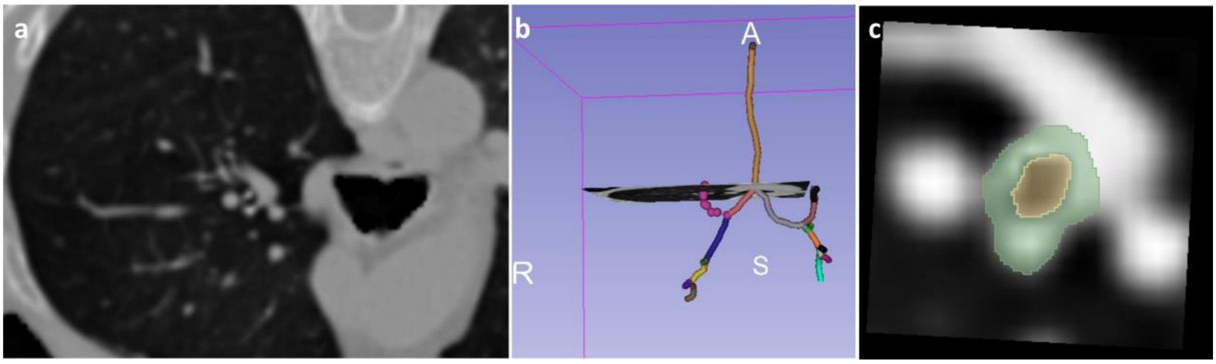

Supplementary Figure E3. Bronchial tree segmentation masks applied on T2w images. (a, b) Registered axial CT images of severe asthma patient centred on right upper lobe bronchus RB1 and left lower lobe bronchus LB10 respectively. (c, d) Registered axial T2w MRI images of the same patient centred on the same bronchi. Visualisation using 3D slicer.

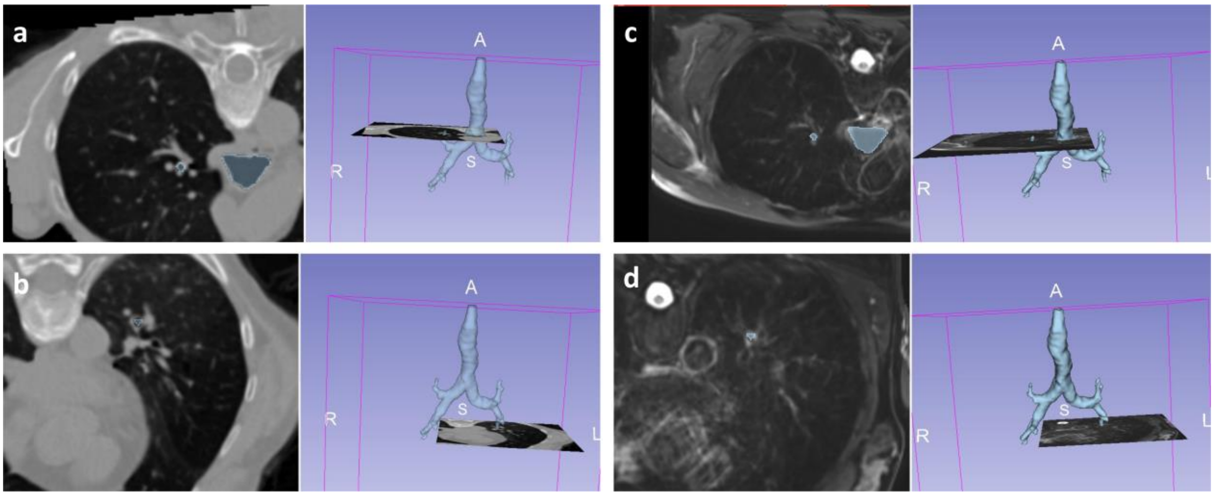

Supplementary Figure E4. Receiver Operator Characteristics Curve of WA% measurements to distinguish between severe asthmatic and non-severe asthmatic patients. The red point represents the best sum of sensitivity and specificity.

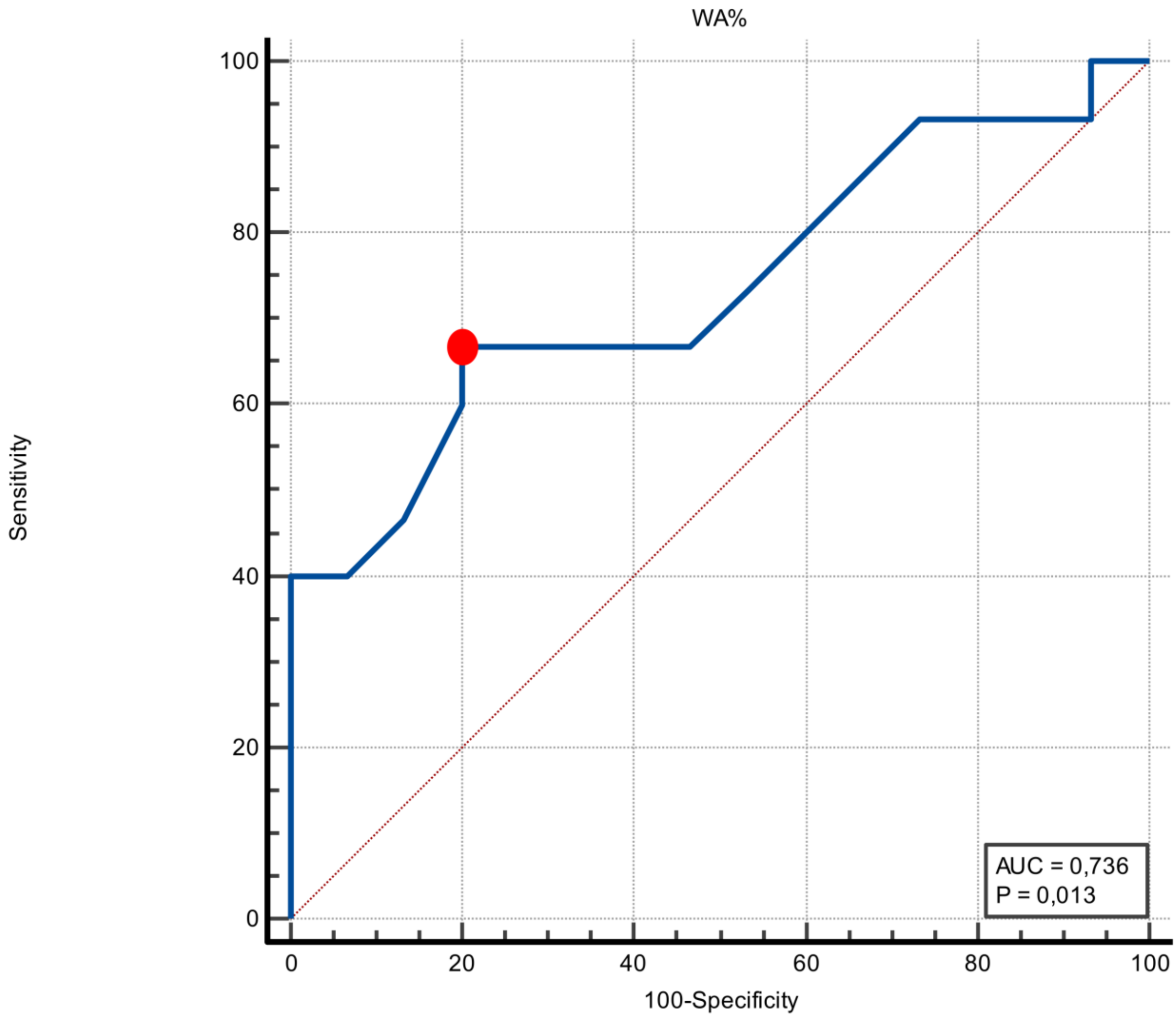

Supplementary Figure E5. Bland Altman plot of BrWall_T2MIS measurements performed by IB and GD.

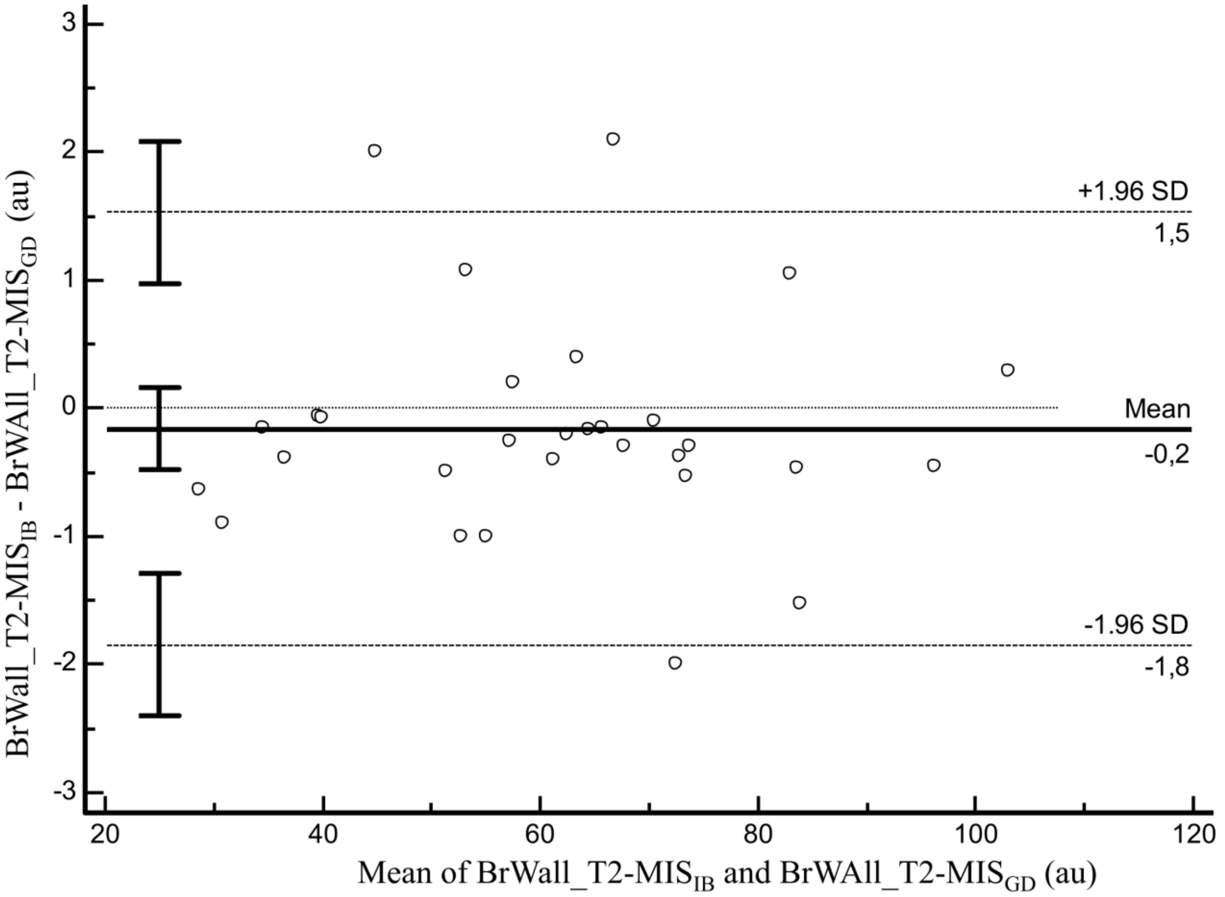

Supplementary Figure E6. Bland Altman plot of BrWall_T2MIS measurements performed twice by IB.

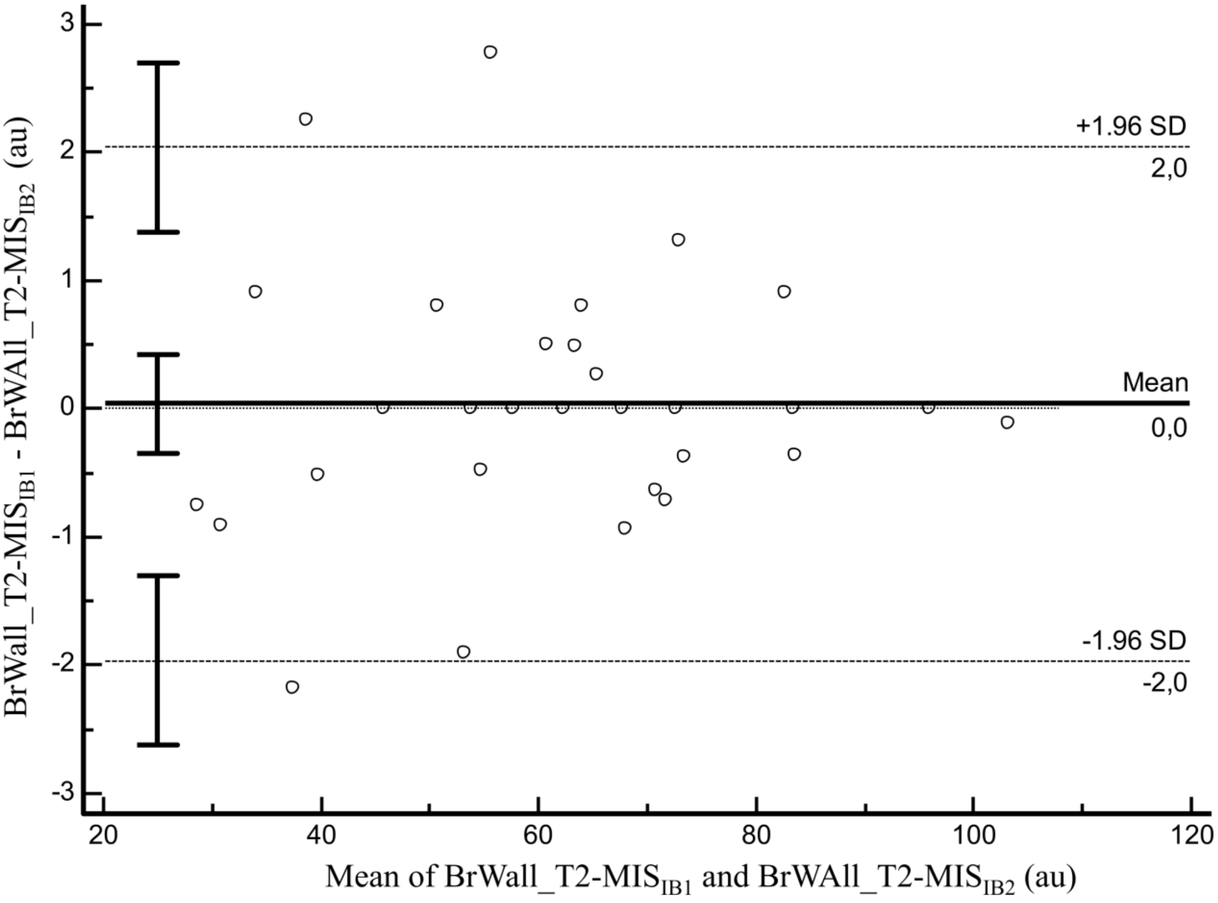

Supplement: Supplementary file 7 — Supplementary MaterialR1 [file 13244_2025_1939_MOESM7_ESM.pdf]
